# Influence of N6-Methyladenosine Modification Gene HNRNPC on Cell Phenotype in Parkinson's Disease

**DOI:** 10.1155/2021/9919129

**Published:** 2021-12-20

**Authors:** Wei Quan, Jia Li, Li Liu, Qinghui Zhang, Yidan Qin, Xiaochen Pei, Jiajun Chen

**Affiliations:** Department of Neurology, China–Japan Union Hospital of Jilin University, No. 126, Xian Tai Road, Changchun, Jilin 130000, China

## Abstract

This study aimed to explore the N6-methyladenosine (m6A) modification genes involved in the pathogenesis of Parkinson's disease (PD) through data analysis of the two data sets GSE120306 and GSE22491 in the GEO database and further explore its influence on cell phenotype in PD. We analyzed the differentially expressed genes and function enrichment analysis of the two sets of data and found that the expression of the m6A-modification gene *HNRNPC* was significantly downregulated in the PD group, and it played an important role in DNA metabolism, RNA metabolism, and RNA processing and may be involved in PD. Then, we constructed the *HNRNPC* differential expression cell line to study the role of this gene in the pathogenesis of PD. The results showed that overexpression of *HNRNPC* can promote the proliferation of PC12 cells, inhibit their apoptosis, and inhibit the expression of inflammatory factors IFN-*β*, IL-6, and TNF-*α*, suggesting that *HNRNPC* may cause PD by inhibiting the proliferation of dopaminergic nerve cells, promoting their apoptosis, and causing immune inflammation. Our study also has certain limitations. For example, the data of the experimental group and the validation group come from different cell types, and the data of the experimental group involve individuals with G2019S *LRRK2* mutations. In addition, due to the low expression of *HNRNPC* in PC12 cells, we used the method of overexpressing this gene to study its function. All these factors may cause our conclusions to be biased. Therefore, more research is still needed to corroborate it in the future.

## 1. Introduction

Parkinson's disease (PD) is the second most common neurodegenerative disease among the elderly [1]. It is characterized by motor dysfunction such as motor retardation, limb tremor, and muscle rigidity and is caused by a lack of dopamine in the midbrain region [2]. There is still a lack of effective treatments to prevent its occurrence and development [3]. Therefore, in-depth exploration of the etiology and pathogenesis of PD and seeking effective treatment measures are currently urgent problems to be solved.

Studies have shown that epigenetic modifications play an important role in the pathogenesis of many neurodegenerative diseases such as Parkinson's disease and Alzheimer's disease [4]. N6-methyladenosine (m6A), as the most common modification of eukaryotic RNAs, refers to the methylation modification on the 6th nitrogen atom of the RNA molecule adenine, which is mainly involved in the transcription, processing, transport, translation, and degradation of mRNA as well as the development and differentiation of cells [5]. It is essential in posttranscriptional regulation [6]. The dynamic changes of m6A can affect gene expression and various RNA signaling pathways, and it has been reported to be associated with the pathogenesis of PD [4, 7–9]. However, we still know little about the relationship between m6A modification and the pathogenesis of PD. The m6A modification requires adenosine methyltransferase (“writers”), demethylase (“erasers”), and RNA binding protein (“readers”) to mediate [7]. These three types of core proteases play an important role in ensuring normal m6A modification [8]. It has been found that the methyltransferase complexes that can catalyze the formation of m6A include *METTL3*, *METTL14*, *WTAP*, *KIAA1429*, etc. [9]. Demethylases mainly include *FTO* and *ALKBH5*. The related enzymes involved in methylation recognition mainly include *YTHDF1-3*, *YTHDC1-3*, *hnRNP*, *eIF3*, etc. [9–11].

In order to explore the modification of genes involved in the regulation of m6 modification, the research group first analyzed two sets of data in the Gene Expression Omnibus (GEO, http://www.ncbi.nlm.nih.gov/geo/) [12] database and found that heterogeneous nuclear ribonucleoprotein C (*HNRNPC*), as a key gene recognized by m6A modification, was significantly downregulated in PD. Then, we further performed analysis by constructing the *HNRNPC* differential expression cell line and found that it plays an important role in the pathogenesis of PD. *HNRNPC* is a member of the heterogeneous nuclear ribonucleoproteins (hnRNPs). As an RNA binding protein, it is considered a “reader” of m6A modification [13]. *HNRNPC* can regulate the nonspecific RNA output, RNA expression, stability, and 3′ end processing and translation of RNA splicing sequences [14] and plays an important role in a variety of cancers and neurodegenerative diseases such as Alzheimer's disease [15]. However, the relationship between *HNRNPC* and PD has not yet been reported. This study proposes that the m6A methylation gene *HNRNPC* is related to the pathogenesis of PD, which provides a theoretical basis for revealing the role of m6A modification in the pathogenesis of PD and provides a new potential target for the treatment of PD.

## 2. Materials and Methods

### 2.1. Data Processing and DEG Screening

We downloaded two sets of data related to Parkinson's disease from the GEO public database: one set is transcriptome data GSE120306, including four PD patients and three healthy individuals. This data set corresponds to RNA-Seq profiles of human iPSC-derived midbrain-patterned astrocytes from 7 donors, including 4 patients with Parkinson's disease who carry the *LRRK2* G2019S mutation, and 3 healthy control individuals. This group serves as the experimental group. In another set of chip data GSE22491, the sample includes ten PD patients and eight healthy controls, and RNAs were extracted from peripheral mononuclear blood cells. The experimental platform is GPL6480 Agilent-014850 Whole Human Genome Microarray 4 × 44K G4112F (Probe Name version). This group serves as a verification group. Then, we used the R software package Oligo [16] (version 1.38.0, http://bioconductor.Org/help/search/index.html?q=oligo/) to perform expression value background correction and expression profile data normalization pretreatment.

We used the classic Bayesian method provided by the limma package (Version 3.30.13, http://www.bioconductor.org/packages/2.9/bioc/html/limma.html) to analyze differentially expressed genes (DEGs) on the two sets of data. We regarded genes with *P* value < 0.05 and log FC absolute value ≥ 0.263 (fold change = 1.2) as DEGs and further identified DEGs related to m6A modification.

### 2.2. Functional and Pathway Enrichment Analysis

We used the differential gene analysis results of the experimental group data set GSE120306 for function and pathway enrichment analysis through clusterProfiler [17] and obtained the GO function and KEGG pathway involved in differential genes. The identification criterion was *P* < 0.05. Then, we performed functional and pathway enrichment analysis on the identified differentially expressed m6A modification genes.

We used the validation group data GSE22491 to verify the expression of DEGs in the experimental group in PD. We took the intersection of the DEGs obtained after data processing of the experimental group and the validation group and displayed them with a Venn diagram. Then, we verified the differential expression of m6A modification genes in the two sets of data, drew box plots of m6A modification genes based on the expression values, and showed their expression between the PD group and the control group in the two sets of data.

### 2.3. Overexpression *HNRNPC* Plasmid Construction

The whole gene synthesis method was used to obtain the ORF sequence of the *HNRNPC* gene with specific sticky ends. The target vector pcDNA3.1-EGFP was digested. The purified synthetic product is connected with the linearized vector, and the ligated product is transformed into bacterial competent cells. The grown single clones are first sequenced and identified, and the sequencing results are compared and analyzed. The sequence is completely correct, which is the successful target gene. Expression plasmid vector was named pcDNA-EGFP-HNRNPC. The constructed overexpression plasmid vector is extracted to obtain a sufficient amount of overexpression vector plasmid.

### 2.4. Cell Culture and Transfection

Rat adrenal medullary pheochromocytoma cells (PC12, undifferentiated) were purchased from Shanghai Chinese Science Cell Bank. The cells were cultured in RPMI-1640 medium containing 1% penicillin-streptomycin double antibody, 5% high-quality fetal bovine serum, and 10% heat-inactivated horse serum. The medium was changed once every 2-3 days, 1 : 2- passaged in a ratio of 1 : 4. Gas phase was as follows: air, 95%; carbon dioxide, 5%, temperature: 37°C.

We inoculated PC12 cells into a 6-well plate, inoculated 5 × 105 cells per well, and performed culture for 24 hours. When they grow to about 80%, we used lipofectamine 2000 for transfection and changed the medium 6 hours after transfection. Cells were harvested for 48 hours after transfection.

### 2.5. QPCR and Western Blotting Detection of Transfection Efficiency

Firstly, total RNA was isolated from transfected cells by TRIzol method. Then RNA was reverse-transcribed into cDNA using cDNA reverse transcription kit (takara, Japan). Finally, fluorescence quantitative PCR was performed using designed specific primers and SYBR green I fluorescent dye detection. The PCR primer sequence is HNRNPC-F: GTCCCCTCTACTCAGTTCCTCAT, HNRNPC-R: TGGAAGAAGATCCCCGTTGT. A total of 30 cycles are set (denaturation: 95°C/2 minutes, annealing: 50°C/2 minutes, extension: 60°C/1 minute).

We used RIPA Lysis Buffer to prepare cell lysates from transfected cells. We placed the lysate on ice for a few minutes and pipetted to fully lyse the cells. Then, we transferred it to a 1.5 mL centrifuge tube and shook vigorously for 30 seconds. Finally, it was centrifuged at 12,000 rpm at 4°C for 15 minutes, and we aspirated the supernatant for subsequent electrophoresis. The electrophoresis was run on an SDS-PAGE gel. The primary antibody was *HNRNPC* (rabbit source, Abcam), and the secondary antibody was goat anti-rabbit IgG (sigma).

### 2.6. CCK8, Flow Cytometry, ELISA, and Transmission Electron Microscope to Detect the Influence of Overexpression of *HNRNPC* on Cell Phenotype

The CCK-8 method was used to calculate cell viability and cell inhibition rate to evaluate the effect of overexpression of *HNRNPC* on cell proliferation. The CCK-8 kit is a rapid detection kit that is widely used in cell proliferation research. Its main component is 2-(2-methoxy-4-nitrophenyl)-3-(4-nitrophenyl)-5-(2,4-disulfobenzene)-2H-tetrazole monosodium salt (WST-8). In the presence of electronic coupling reagents, WST-8 can be reduced by dehydrogenase in the mitochondria to produce a highly water-soluble orange-yellow formazan product. The intensity of the color after the reaction is directly proportional to the proliferation of the cells. Using a microplate reader to measure the OD value at 450 nm wavelength can reflect the number of viable cells. The cells were seeded in a 96-well plate for transfection, and cell proliferation was detected at 24 h, 48 h, and 72 h after transfection. After the treatment is completed, we added 10 *μ*L CCK-8 to each well, incubated at 37°C for 1–4 h, and measured the absorbance (OD) at 450 nm with a microplate reader. Cell viability (%) = [A (experimental group)-A (blank group)]/[A (control group)- A (blank group)] × 100%. Cell inhibition rate (%) = [A (control group)-A (experimental group)]/[A (control group)-A (blank group)] = 1 − cell viability (A (experimental group): absorbance value of treated cells and CCK solution; A (blank group): absorbance value of wells with culture medium and CCK solution but no cells; A (control group): untreated absorbance value of cells and CCK solution).

The Annexin V-PE/ 7-AAD flow cytometry kit was used to perform double staining of Annexin V-PE and 7-AAD on each group of specimens, and then the apoptosis was detected by flow cytometry in order to evaluate the effect of overexpression of *HNRNPC* on cell apoptosis.

The expression levels of IFN-*β*, IL-6, and TNF-*α* in the specimens were determined using rat interferon-*β* (IFN-*β*) ELISA kit, rat interleukin 6 (IL-6) ELISA kit, and rat tumor necrosis factor alpha (TNF-*α*) ELISA kit. The kit uses the double-antibody Sandwich method to determine the levels of various inflammatory factors in the specimen, and the OD value is measured with a microplate reader at a wavelength of 450 nm, and then the concentration of various inflammatory factors in each sample is calculated in order to evaluate the effect of overexpression of *HNRNPC* on the expression of inflammatory factors in cells.

Transmission electron microscopy was used to observe the autophagy after overexpression of *HNRNPC*. After collecting the cells, we adjusted the concentration to 1∼5 × 105/mL, performed centrifuge at 3000 rpm for 3 min, and removed the supernatant. We doubled fixation with 4% glutaraldehyde and 1% osmium tetrachloride. We performed acetone dehydration step by step and added acetone-EPON812 package at room temperature. Embedding agent was 3∼4 mL/30 min, and pure embedding agent was 1∼2 mL/2h for complete embedding. It was left baking in a 60°C oven for 24 h to solidify into embedding lumps. The embedded hard block was cut into semi-thin sections with a thickness of about 1 μm using an ultrathin microtome and dry them in an oven. Then it was dyed with methylene blue dye solution and composite dye (0.25% sodium borate: 0.5% basic fuchsin 1 : 1), cut into 50 nm ultrathin sections again, immerse, in 0.45% Fonnvar solution prepared by chloroform, taken out immediately to promote film formation, and stained with saturated uranyl acetate solution and lead citrate staining solution at room temperature for 10 min and 12 min, respectively. The filter paper was blotted, and the sample cells were observed by transmission electron microscope.

### 2.7. Statistical Analysis

Statistical analyses were performed using GraphPad Prism 8.0. Normally distributed data were analyzed using a two-tailed Student's *t*-test when comparing two groups, or a one-way or two-way ANOVA with Bonferroni's multiple comparison posttest when comparing more than two groups. For nonnormally distributed data (Shapiro-Wilk test, *P* < 0.05), a nonparametric two-tailed Mann-Whitney *U* test was used when comparing two groups, or a Kruskal-Wallis test with Dunn's multiple comparison posttest when assessing more than two groups. Differences with *P* < 0.05 were considered statistically significant. All data are presented as the mean ± Standard Error of the Mean (SEM) if not mentioned otherwise.

## 3. Results

### 3.1. DEG Analysis

**Figure 1:**
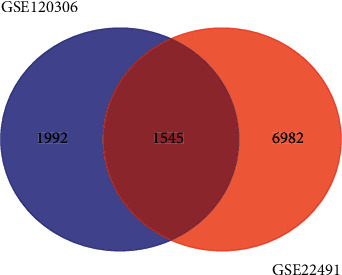
Venn diagrams of DEGs in the experimental group and the verification group.

DEGs were analyzed for the two groups of data according to PD and the healthy control group. The statistical results of the difference analysis of the two groups of data are shown in Table 1. The intersection of the two sets of data for DEGs is shown in Figure 1. The list of the same m6A modification genes is shown in Table 2. It can be seen that, in the two sets of data, the same genes related to m6A modification and their expressions are consistent: the ENST00000556226 transcript of *HNRNPC* in GSE120306 and the A_32_P142028 and A_24_P178423 probes in GSE22491 are all downregulated.

### 3.2. Functional Enrichment Analysis

**Figure 2:**
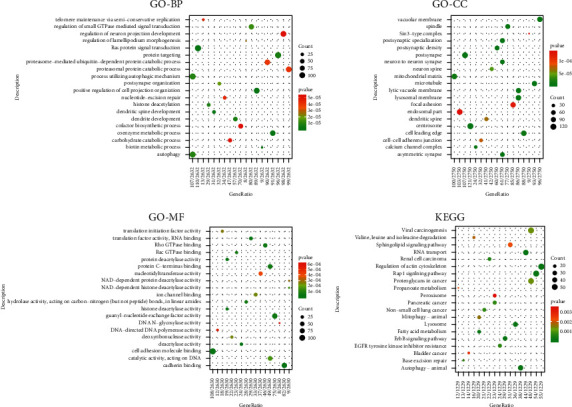
Bubble chart of GO-KEGG enrichment results. The more the genes in each enrichment item, the bigger the bubble, and the *P* value from high to low corresponds to the color from red to green.

**Figure 3:**
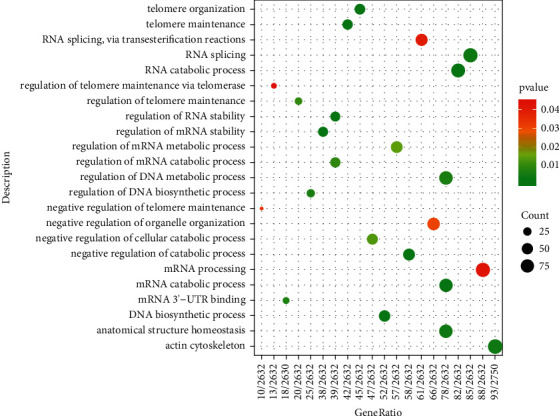
GO analysis involving *HNRNPC*. The more the genes in each enrichment item, the larger the bubble, and the *P* value from high to low corresponds to the color from red to green.

The results of DEGs in the experimental group data set GSE120306 were enriched and analyzed, and the GO function and KEGG pathway involved in differential genes were obtained, as shown in Figure 2. From the enrichment results in Figure 2, the GO entries that *HNRNPC* participates in are identified (this gene is not enriched in KEGG), as shown in Figure 3. According to the GO entry, *HNRNPC* is mainly involved in the functions of DNA metabolism, RNA metabolism, and RNA processing.

### 3.3. Validation Analysis

**Figure 4:**
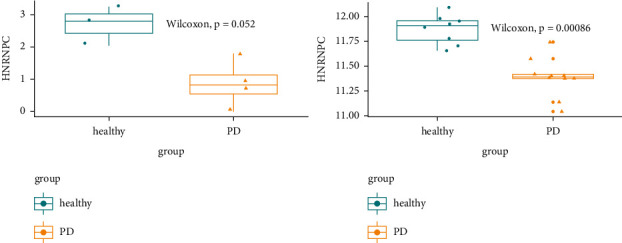
The expression of *HNRNPC* gene in two sets of data sets.

Using the expression values of the two sets of data, a box plot was drawn against the expression values of the transcripts and probes of the *HNRNPC* gene in Table 2, as shown in Figure 4. It can be seen from the figure that the expression of the *HNRNPC* gene is downregulated in both sets of data PD groups. Statistical analyses were performed using GraphPad Prism 8.0.

### 3.4. Overexpression of *HNRNPC* Promotes PC12 Cell Proliferation

**Figure 5:**
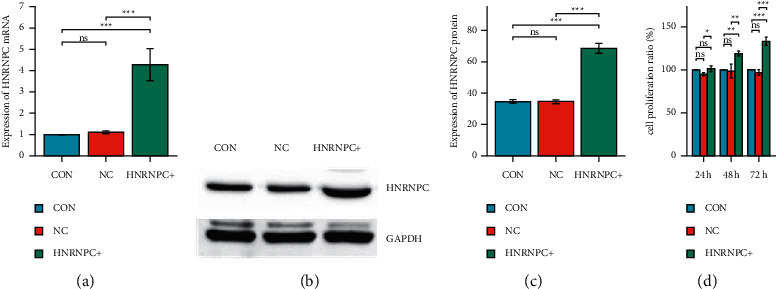
The effect of overexpression of *HNRNPC* on the proliferation of PC12 cells. (a) Detection of HNRNPC mRNA expression in each group by qPCR. (b, c) Detection of HNRNPC protein expression in each group by western blotting. (d) Detection of cell proliferation ratio in each group by CCK-8. CON: control group, NC: empty plasmid group, HNRNPC+: overexpression *HNRNPC* group.

In view of the low expression of *HNRNPC* in PC12 cells, it was decided to use the method of overexpression of *HNRNPC* to interfere with its expression in PC12 to explore the function of *HNRNPC* in nerve cells. First, we transfected the successfully constructed *HNRNPC* overexpression plasmid into PC12 cells. The transfection results are shown in Figures 5(a)–5(c). Compared with the normal group and the empty group, the *HNRNPC* mRNA and protein expressions in the overexpression *HNRNPC* plasmid group were significantly increased.

In order to explore the effect of overexpression of *HNRNPC* on the proliferation of PC12 cells, we transfected the *HNRNPC* overexpression plasmid or empty plasmid into PC12 and used CCK-8 to test the cell proliferation capacity at 24, 48, and 72 hours after transfection. The results are shown in Figure 5(d). Compared with the normal group and the empty group, the cell proliferation ability of the *HNRNPC* plasmid overexpression group increased, and the increase of the cell proliferation ability became more significant as time passed. Statistical analyses were performed using GraphPad Prism 8.0.

### 3.5. Overexpression of *HNRNPC* Inhibits PC12 Cell Apoptosis

**Figure 6:**
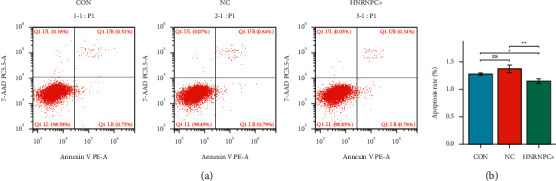
The effect of overexpression of *HNRNPC* on PC12 cell apoptosis. CON: control group, NC: empty plasmid group, HNRNPC+: overexpression *HNRNPC* group.

In order to explore the effect of overexpression of *HNRNPC* on PC12 cell apoptosis, we transfected the *HNRNPC* overexpression plasmid or empty plasmid into PC12 and used flow cytometry to detect the proportion of apoptotic cells in each group of samples. The results are shown in Figures 6(a) and 6(b). Compared with the normal group and the empty group, the percentage of apoptosis in the *HNRNPC* plasmid overexpression group was reduced and the difference was statistically significant. Statistical analyses were performed using GraphPad Prism 8.0.

### 3.6. Overexpression of *HNRNPC* Inhibits the Expression of Inflammatory Factors

**Figure 7:**
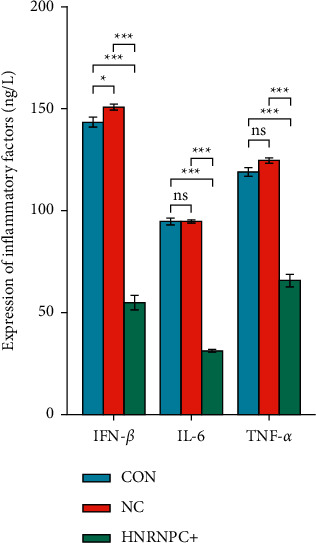
The effect of overexpression of *HNRNPC* on the expression of inflammatory factors in each group. CON: control group, NC: empty plasmid group, HNRNPC+: overexpression *HNRNPC* group.

In order to explore the effect of overexpression of *HNRNPC* on the expression of inflammatory factors in PC12 cells, we transfected *HNRNPC* overexpression plasmid or empty plasmid into PC12 and used ELISA to detect the expression levels of IFN-*β*, IL-6, and TNF-*α* in each group of samples. The results are shown in Figure 7. Compared with the normal group and the empty group, the expression levels of IFN-*β*, IL-6, and TNF-*α* in the *HNRNPC* plasmid overexpression group were significantly reduced. Statistical analyses were performed using GraphPad Prism 8.0.

### 3.7. Overexpression of *HNRNPC* Has No Effect on Autophagy in PC12 Cells

**Figure 8:**
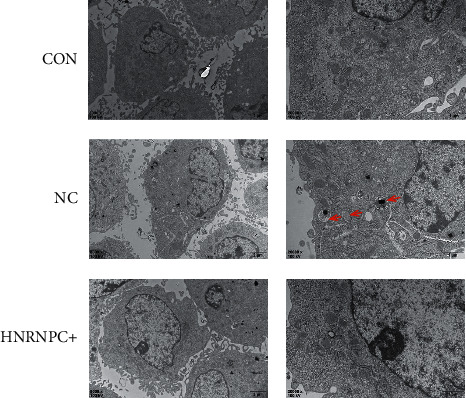
The effect of overexpression of *HNRNPC* on autophagy in PC12 cells. The red arrow in the figure is the autophagosome. CON: control group, NC: empty plasmid group, HNRNPC+: overexpression *HNRNPC* group.

In order to explore the effect of overexpression of *HNRNPC* on autophagy in PC12 cells, we transfected the *HNRNPC* overexpression plasmid or empty plasmid into PC12 and observed the formation of autophagosomes in the cells by transmission electron microscope. The results are shown in Figure 8. The formation of autophagosomes was seen in the empty plasmid transfection group, but no autophagosomes were observed in the control group and the *HNRNPC* overexpression group, indicating that *HNRNPC* may have nothing to do with nerve cell autophagy.

## 4. Discussion

PD is a common movement disorder in the elderly, and about 1.5% to 2.0% of people over 60 years of age are affected by it [18]. Due to the unclear pathogenesis of PD and the lack of effective clinical treatment methods, it is of great significance to explore its pathogenesis. In recent years, m6A modification, as a common mRNA modification, has attracted more and more attention from researchers due to its key modification effect on a variety of cell pathological processes [19]. m6A is highly expressed in the brain. The m6A modification of mRNA has a wide range of effects on the nervous system and plays an important role in the self-renewal of neural stem cells, brain development, learning and memory, and synaptic growth [20]. Previous studies have shown that m6A modification can participate in midbrain dopaminergic signaling and is related to the pathogenesis of PD [4]. Qiu et al. [21] found that five m6A-related SNPs (rs75072999 of *GAK*, rs1378602, rs4924839 and rs8071834 of *ALKBH5*, and rs1033500 of *C6orf10*) are related to changes in gene expression in PD through expression quantitative trait loci (eQTL) analysis. Chen et al. [22] found that the PD cell model and rat model induced by 6-OHDA showed that m6A modification of mRNA was downregulated. The reduction of m6A could induce the expression of *NMDA* receptor 1, increasing oxidative stress and calcium influx, resulting in dopaminergic neuron apoptosis. Recently, *FTO* gene inactivation has also been shown to impair dopamine receptor-dependent neuronal activity and behavioral response control [23]. These evidences all suggest that m6A modification disorders are involved in the pathogenesis of PD. However, the relationship between the two is still unclear. This study uses a combination of bioinformatics analysis and experimental verification to explore the role of m6A modification genes in the pathogenesis of PD and opens up a new perspective for revealing the pathogenesis of PD.

Heterogeneous nuclear ribonucleoproteins (hnRNPs) are a family of multifunctional protein molecules. As “readers” of m6A, they have been proven to play an important role in the process of m6A recognition [24]. According to reports, hnRNPs are involved in a series of important processes such as the splicing of mRNA precursors, mRNA nucleocytoplasmic transport, translation, and degradation and plays a vital role in the incidence of neurodegenerative diseases such as AD, amyotrophic lateral sclerosis (ALS), and frontotemporal lobe degeneration (FTLD) [15, 25, 26]. Mutations in the prion-like domains of hnRNP A1 and A2/B1 are thought to be the cause of ALS and FTLD [27]. The family member *HNRNPC* can participate in the pathogenesis of AD by intervening in the translation of amyloid precursor protein (APP) to affect *β*-amyloid protein deposition [28]. Based on the above evidence, we speculate that members of the hnRNPs related to m6A modification may also be involved in the pathogenesis of PD. Our study found that the expression of *HNRNPC*, a member of the hnRNPs, was significantly downregulated in the PD group compared with the control group, and the difference was statistically significant, suggesting that *HNRNPC* may be involved in the pathogenesis of PD.

*HNRNPC* and hnRNP A1, A2, B1, and B2 are the first members of the hnRNPs to be discovered, and they are collectively called core hnRNP [29]. It has two alternative splice variants (HNRNPC1 and C2) [30] and is mainly located in the nucleus, where it mediates the transfer of multiple RNA transcripts and proteins between the nucleus and the cytoplasm [31]. *HNRNPC* contains only one RNA binding domain (RRM), which must interact with its RNA target after being oligomerized into a tetramer [32]. It is considered as a “reader” of m6A and can selectively identify m6A mRNA sites that mediate mRNA degradation [33]. Studies have shown that *HNRNPC* plays an important role in the process of RNA metabolism such as mRNA recognition and classification, alternative splicing of mRNA precursors, mRNA transport, and stability regulation, and it also participates in mRNA transcription and translation regulation [34, 35]. Our GO analysis results also suggest that *HNRNPC* plays an important role in DNA metabolism, RNA metabolism, and RNA processing and may be involved in the pathogenesis of PD, which is consistent with the previously reported results. In order to further confirm the role of *HNRNPC* in the pathogenesis of PD, we deeply explored the specific mechanism of *HNRNPC* in PD by constructing a *HNRNPC* differential expression cell line.

Previous studies have shown that *HNRNPC* is highly expressed in lung cancer [36], gastric cancer [37], ovarian cancer [38], and other tumor cells. Silencing *HNRNPC* can enhance etoposide-induced glioblastoma apoptosis [39]. HNRNPC1 and C2 can regulate the expression of X-chromosome-linked inhibitor of apoptosis (XIAP) by specifically enhancing the XIAP translation of internal ribosome entry site (IRES), thereby affecting the process of apoptosis [40]. *HNRNPC* can also affect cell proliferation by regulating the translation of c-myc mRNA in a cell cycle-dependent manner by combining with IRES [41]. Wu et al. found that knocking out *HNRNPC* can inhibit breast cancer cell proliferation by promoting IFN-*β* expression [14]. However, research on *HNRNPC* and PD is rarely reported. Considering that a large number of previous studies have confirmed [42–46] that apoptosis, autophagy, and immune inflammatory mechanisms are crucial in the pathogenesis of PD, as well as confirming the role of *HNRNPC* in the process of cell proliferation and apoptosis, our study initially explored the role of *HNRNPC* in the pathogenesis of PD based on the above phenotype. Our results show that, compared with the control group, overexpression of *HNRNPC* can promote the proliferation of dopaminergic nerve cells PC12 cells, inhibit their apoptosis, and inhibit the expression of the inflammatory factor IFN-*β*, which is consistent with the above research results. Our results also suggest that overexpression of *HNRNPC* can inhibit the expression of inflammatory factors IL-6 and TNF-*α*, and these two inflammatory factors have been confirmed to be involved in the pathogenesis of PD [47]. In addition, taking into account the role of autophagy in the pathogenesis of PD and the impact of other members of hnRNPs on autophagy, for example, hnRNPA1 can promote the expression of autophagy-related gene 6 (ATG6) by binding to the 3′ UTR of its mRNA, thereby playing an important role in the pathogenesis of colorectal cancer [48], and hnRNP K can participate in the formation of adriamycin resistance in acute myeloid leukemia by regulating autophagy [49]. Our research group observed the effect of overexpression of *HNRNPC* on autophagy in PC12 cells through transmission electron microscopy. Our results showed that the formation of autophagosomes was seen in the empty plasmid transfection group, but no autophagosomes were observed in the control group and the *HNRNPC* overexpression group. Therefore, our results suggest that overexpression of *HNRNPC* may have no effect on autophagy in PC12 cells. However, it is still one-sided to draw conclusions based on the results of electron microscopy, and more autophagy-related experiments are needed to prove it in the future.

In conclusion, our results show that the expression of the m6A modification gene *HNRNPC* is downregulated in PD; overexpression of *HNRNPC* can promote PC12 cell proliferation, inhibit its apoptosis, and inhibit the expression of inflammatory factors IFN-*β*, IL-6, and TNF-*α*, suggesting that *HNRNPC* may cause PD by inhibiting the proliferation of dopaminergic nerve cells, promoting their apoptosis, and causing immune inflammation. Our research is the first to propose that the m6A modification gene *HNRNPC* is related to the pathogenesis of PD, provides a theoretical basis for revealing the role of m6A modification in the pathogenesis of PD, and provides a new potential target for the treatment of PD. Our study also has certain limitations. For example, the data of the experimental group and the validation group come from different cell types, and the data of the experimental group involve individuals with G2019S *LRRK2* mutations. In addition, due to the low expression of *HNRNPC* in PC12 cells, we used the method of overexpressing this gene to study its function. All these factors may cause our conclusions to be biased. Therefore, more research is still needed to corroborate it in the future.

## Figures and Tables

**Table 1:** Statistics of DEGs.

DEGs	Up	Down	All
GSE120306 (test group)	1342	2355	3697
GSE22491 (verification group)	7707	1002	8709

**Table 2:** The m6A modification genes in the two groups of DEGs.

DataSet	Symbol	Trans	logFC	*P* value
GSE120306	HNRNPC	ENST00000556226	−1.837	0.013486
	HNRNPC	ENST00000555914	10.13	1.18*E* − 02
	HNRNPC	ENST00000555176	0.698	2.07*E* − 02
	FTO	ENST00000637001	−3.665	2.05*E* − 03

		ID		
GSE22491	HNRNPC	A_32_P142028	−0.45375067	9.58*E* − 05
	HNRNPC	A_24_P178423	−0.52154436	0.00016
	WTAP	A_23_P215037	−0.77027173	3.01*E* − 07
	ALKBH5	A_23_P416169	−0.72699349	6.55*E* − 06
	WTAP	A_32_P219368	−0.50259394	0.00018
	METTL14	A_23_P110243	−0.60307577	0.000302
	ALKBH5	A_23_P153050	−0.42392063	0.0016
	HNRNPDL	A_23_P213153	−0.38698579	0.00171
	HNRNPDL	A_24_P9090	−0.38731852	0.00252
	WTAP	A_23_P371155	−0.44288286	0.00795
	EIF3A	A_23_P86550	−0.3217432	0.0145
	METTL3	A_23_P54064	−0.45666822	0.0222

## Data Availability

In this study, the gene expression datasets analyzed were obtained from the GEO database (https://www.ncbi.nlm.nih.gov/geo/). After a careful review, we choose the transcriptome data GSE120306 as the experimental group and another set of chip data GSE22491 as a verification group. The experimental platform is GPL6480 Agilent-014850 Whole Human Genome Microarray 4 × 44K G4112F (Probe Name version). The data are freely available online.
